# Learning about cardiac arrest from 'Dr. Google': a pre- and peri-pandemic infodemiology study in Nigeria

**DOI:** 10.11604/pamj.2022.42.22.34249

**Published:** 2022-05-10

**Authors:** Tonia Chinyelu Onyeka, Ijeoma Uchenna Itanyi, Hilary Uchenna Ezugwu, Matthew Allsop

**Affiliations:** 1 Department of Anaesthesia/Pain and Palliative Care Unit, College of Medicine, University of Nigeria Ituku-Ozalla Campus, Enugu, Enugu State, Nigeria,; 2 Center for Translation and Implementation Research, College of Medicine, University of Nigeria, Ituku-Ozalla Campus, Enugu, Enugu State, Nigeria,; 3 Department of Community Medicine, College of Medicine, University of Nigeria, Ituku-Ozalla Campus, Enugu, Enugu State, Nigeria,; 4 Department of Anaesthesia, University of Nigeria Teaching Hospital, Ituku-Ozalla, Enugu, Enugu State, Nigeria,; 5 Academic Unit of Palliative Care, Leeds Institute of Health Sciences, University of Leeds, Leeds, United Kingdom

**Keywords:** Cardiac arrest, heart attack, heart arrest, health promotion, Google

## Abstract

**Introduction:**

the coronavirus pandemic and associated lockdowns restricted movement with non-essential hospital trips discouraged to prevent spread of the virus. Disruption of medical services can lead to increased seeking of medical advice and symptom management online. With COVID-19 known to worsen existing cardiovascular disease or precipitate a new one, we sought to explore online search trends of the Nigerian public regarding cardiac events before and during the COVID-19 pandemic.

**Methods:**

using Google Trends™, relative search volume for the terms 'cardiac arrest', 'heart attack', and 'heart arrest' were analyzed for the periods 27^th^ February to 30^th^ September in 2019 and 2020 respectively. Descriptive statistics, Mann-Whitney U test for relative search volume, search terms comparison in both years and Kendall´s correlation coefficient for examining relationships between time frames and search terms were used.

**Results:**

searches for terms 'heart attack' (p<0.001) and 'heart arrest' (p=0.01) were higher in 2020 compared to 2019, with a correlation between searches for 'cardiac arrest' and 'heart arrest' (p<0.001) and between 'heart attack' and 'heart arrest' (p=0.01). There was a strong positive correlation between search for 'heart attack' in 2019 and 2020 (tau b=0.35, p<0.001); and a moderate positive correlation for 'heart arrest' (tau b=0.13, p=0.01).

**Conclusion:**

increased online activity relating to cardiac arrest was recorded during the early months of the pandemic when compared to the year prior. Notable increases in search activity aligned with the timing of heart-related illnesses and deaths of Nigerian celebrities during the pandemic. Further understanding of health-related online search activity in Nigeria could inform the development of health promotion interventions and support health-related information seeking for cardiovascular diseases.

## Introduction

Cardiac arrest (CA) is a major public health concern [1] being the third leading cause of death in adults [2] and the primary cause of sudden non-traumatic death in children and teenagers worldwide [3]. It refers to the sudden loss of heart function leading to unresponsiveness and the absence of normal breathing and circulation which necessitates the use of cardiopulmonary resuscitation (CPR) to rapidly deliver oxygen to vital organs in order to revive the individual [4]. The incidence of CA and survival from the condition vary globally, ranging from 28.3 per 100,000 person-years, 54.6 per 100,000 person-years and 84 per 100,000 person-years in Asia, North America and Europe respectively [5]. The incidence of CA in sub-Saharan Africa (SSA) generally and in Nigeria in particular is unknown due to improper record-keeping [6]. With the high burden of noncommunicable diseases (NCDs) in low- and middle-income countries (LMICs) [7], the majority of deaths from CA in these countries are a result of cardiovascular diseases such as hypertension and coronary heart disease [8] and these deaths have been predicted to rise further by 2030 [7,9]. One way to reduce the burden of NCDs in LMICs is through public health communication strategies [10]. People living in LMICs in SSA face barriers to healthcare including high out-of-pocket expenses, erratic electricity supply, high electricity tariffs as well as unreliable and slow internet connection, but have well established online health-seeking habits [11-13].

During the coronavirus disease outbreak of 2019 (COVID-19), restrictions of movement instituted during the pandemic resulted in increased screen times, increased sitting time and increased online presence [14]. Subsequently, online searches for medical information increased as most trips to the hospital were discouraged to prevent the spread of the virus [15,16]. Nigeria was not exempt as the restriction in movement occasioned by curfews and lockdown led to a reduction in access to health facilities and may have led to increased use of the internet for health information. The COVID-19 pandemic necessitated several control measures to contain the spread of the virus. These measures included social distancing/physical distancing, regular hand-washing and use of hand sanitizers, face-masks and face shields [17]. In all the thirty-six states of the Nigerian federation and the country´s capital, Abuja, a ban on public gatherings of more than twenty persons came into effect, with a gradual shift to a complete lockdown that saw government offices, schools, churches and businesses closed for three months [18]. The measure of lockdown resulted in lifestyle modifications such as increased physical inactivity and an increase in internet use for remote working, remote schooling and entertainment [14].

Google, via the internet, has been known to be a source of information for the general public as well as a guide for decision-making especially as it relates to health information [19], even though it is fraught with inaccurate and conflicting information [20]. There is little knowledge regarding the impact of restrictions on hospital or healthcare access on online health information seeking [21]. Disruption of preventive and restorative health services, medicine and medical supplies stock-outs have been identified as fall-outs of restrictive social conditions such as pandemics [22]. Some populations have been known to heavily rely on the internet, the latter serving as a proxy measure for their health needs during the COVID-19 lockdown [21]. Within Nigeria, changes in patterns of internet-based heath-seeking behavior during COVID-19 lockdowns have not been reported. Understanding online health-seeking behaviors for specific conditions can be used to determine the demand for information and guide approaches to how it is provided. This study aimed to determine the online interest of the Nigerian public by examining and comparing their online search trends of a health condition, cardiac arrest (CA) across two time points; pre-pandemic and peri-pandemic.

## Methods

The use of Google to search for information relating to cardiac arrest in Nigeria was carried out using Google Trends™. Google Trends™ is a free online tool belonging to Google Inc. that enables near real-time examination of keyword search terms using Google (for example, via web browsers, or on a mobile phone) relative to the total search volume over a specified time period. This metric is typically used as a proxy for the public´s interest [23]. When a term or a set of terms are imputed on Google Trends™, a set of normalized data referred to as the Relative Search Volume (RSV) is represented as a line graph showing the levels of interest or disinterest in the terms over a period of time [24]. The RSV is displayed as a number between 0 and 100, with the latter representing the peak value and the value of 0 signifying that the available data is too low to be quantified. In this study, the following terms were searched for on Google Trends™: “cardiac arrest”, “heart attack”, “heart arrest”, “cardiopulmonary resuscitation”, “basic life support”, and “heart stoppage”. The choice of search terms was guided by common keywords in the cardiac arrest literature and the terms were not used in combination with each other.

The following filters were applied: country - Nigeria; period 27^th^ February to 30^th^ September in 2019 and 27^th^ February to 30^th^ September in 2020; query category - all categories; type of search - web search. The query, “All” was chosen as the query category to reflect all types of searches, for example, health, social or media. Dates were determined as 27^th^ February, 2020 which aligned with when the first case of COVID-19 was identified in Nigeria, to 30^th^ September, 2020, before the study started, which was compared to the same time period in 2019. Data was downloaded on October 18, 2020. Searches utilized the keywords devoid of quotation marks or conjunctions. “Heart stoppage” yielded no results, while the terms “cardiopulmonary resuscitation” and “basic life support” yielded scanty results for only one sub-region, Lagos State, which was not representative of the country hence they were not analyzed. Relative search volumes (RSV) for the terms “cardiac arrest”, “heart attack”, and “heart arrest” were analyzed nationally for the specified periods, 27^th^ February to 30^th^ September in 2019 and 2020 respectively. Sub-regional data were analysed for “cardiac arrest” and “heart attack" but not for “heart arrest” because Google Trends™ reported there was insufficient data to analyse sub-regional statistics for the latter. The daily times series RSV was analyzed using Microsoft Office Excel and Stata version 11.

Time series line plots were used to examine trends of all three search terms analyzed. Mann-Whitney U test was used to compare RSV of each search term in both years. Kendall´s correlation coefficient was used to examine the correlation of RSV between search terms and the correlation of searches in the two search periods. For all analyses, p-value of <0.05 was considered statistically significant. Study reporting is aligned with the Nuti checklist (Annex 1) for studies using the Google Trends™ platform [25] as well as the STROBE checklist for observational cross-sectional studies [26].

## Results

**Figure 1:**
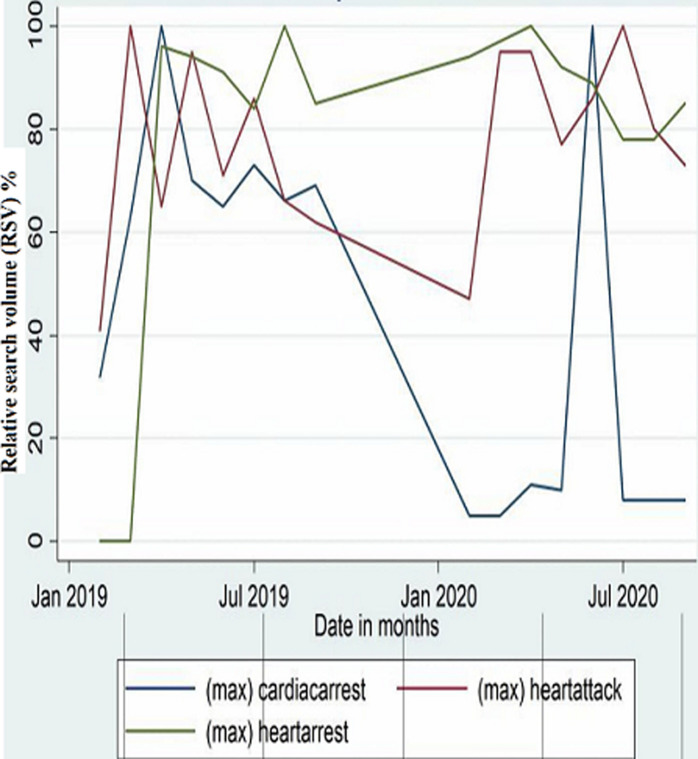
maximum monthly RSVs for 2019 and 2020

**Figure 2:**
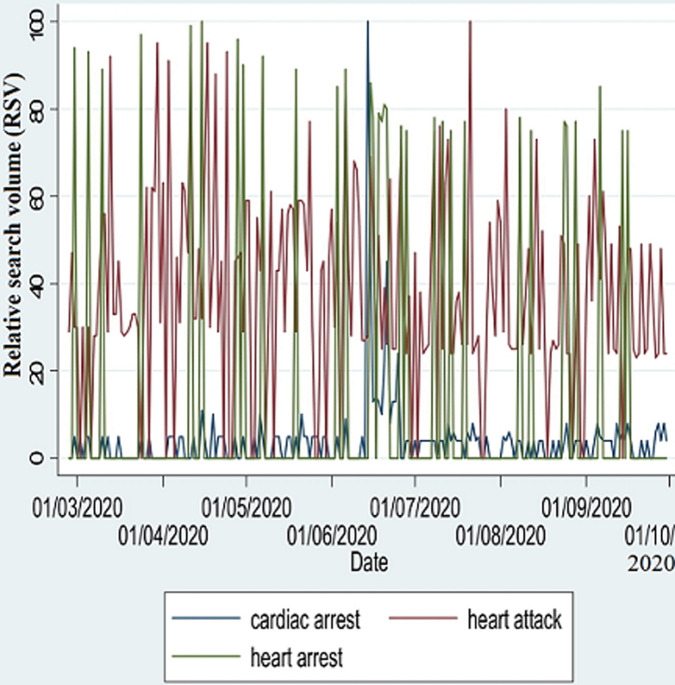
time-series plot of daily relative search volumes for 2020

**Figure 3:**
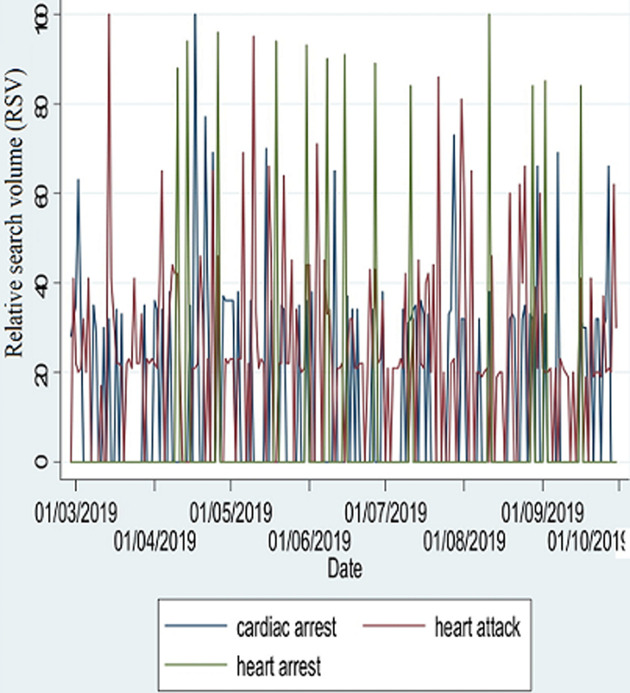
time-series plot of daily relative search volumes for 2019

The peak search volumes are shown in Figure 1. In 2019, the search term, “heart arrest”, had the highest number of peaks (RSV of >90), followed by “heart attack”. “Cardiac arrest” was noted to have had only one peak. Maximum RSV (100) was observed on 14^th^ March for “heart attack”, 17^th^ April for “cardiac arrest”, and 11^th^ August for “heart arrest”. In 2020, the maximum RSV (100) were on 15^th^ April for “heart arrest”, 14^th^ June for “cardiac arrest”, and 21^st^ July for “heart attack”. The search term, “heart arrest” had the highest number of peaks (RSV of >90), followed by “heart attack”, and these were observed mostly in the months of February, March and April. “Cardiac arrest” was observed to be mostly stationary, with one peak observed in June 2020. Searches for “cardiac arrest” and related terms were higher in the peri-pandemic period (Figure 2) than in the pre-pandemic period (Figure 3).

For the sub-regional search statistics, the term, “cardiac arrest” was searched for in nine states of the country in 2019, and in 14 states in 2020 (Table 1). Searches for “heart attack” occurred in seven states in 2019 and in eight states in 2020; All searches for “heart arrest” in 2019 and 2020 were in Lagos State. Searches for “heart attack" (Z for rank sum=-8.57; p<0.001) and “heart arrest” (Z for rank sum=-2.82; p=0.01) were higher in 2020 compared to 2019 (Table 2). There was no correlation between search for “cardiac arrest” and “heart attack” (tau b=-0.01; p=0.78), but there was a correlation between search for “cardiac arrest” and “heart arrest” (tau b=0.16; p<0.001) and between “heart attack” and “heart arrest” (tau b=0.10; p=0.01) (Table 3). There was a strong positive correlation between search for “heart attack” in 2019 and 2020 (tau b=0.35, p<0.001); and a moderate positive correlation for “heart arrest” (tau b=0.13, p=0.01) in 2019 and 2020 (Table 4).

**Table 1:** Google Trends™ search results by state for 2019 and 2020

Search term	2019		2020	
	**State**	**RSV**	**State**	**RSV**
Cardiac arrest	Osun	100	Ogun	100
	Akwa Ibom	82	Oyo	94
	Kaduna	79	Osun	85
	Anambra	62	Kaduna	85
	Edo	43	Akwa Ibom	71
	Rivers	39	Anambra	67
	Oyo	27	Kwara	61
	Federal capital territory	16	Imo	61
	Lagos	14	Delta	60
			Lagos	52
			Enugu	48
			Edo	43
			Federal capital territory	40
			Rivers	24
	**State**	**RSV**	**State**	**RSV**
Heart attack	Delta	100	Anambra	100
	Oyo	91	Ogun	75
	Ogun	71	Oyo	74
	Federal capital territory	57	Delta	72
	Lagos	53	Lagos	62
	Rivers	44	Federal capital territory	61
	Edo	43	Rivers	59
			Edo	48


RSV: relative search volume

**Table 2:** differences in relative search volume of search terms in 2019 and 2020

Variable	Rank sum	Z	P-value
**Cardiac arrest**			
2019	47117.5	0.21	0.85
2020	46843.5		
**Heart attack**			
2019	35754	-8.57	<0.001
2020	58207		
**Heart arrest**			
2019	44926	-2.82	0.01
2020	49035		

**Table 3:** correlation of different search terms

Search terms	Tau a	Tau b	P-value
Cardiac arrest and heart attack	-0.0083	-0.0104	0.78
Cardiac arrest and heart arrest	0.0596	0.1636	<0.001
Heart attack and heart arrest	0.0424	0.0985	0.01

**Table 4:** correlation of searches by year (2019 and 2020)

Variable	Tau a	Tau b	P-value
Cardiac arrest	-0.0052	-0.0090	0.84
Heart attack	0.2377	0.3457	<0.001
Heart arrest	0.0416	0.1335	0.01

## Discussion

This study, which aimed at identifying the online search trends of the Nigerian public concerning cardiac arrest, was able to identify changes in search trends during both the pandemic and pre-pandemic period relating to cardiac arrest. Findings indicate that the popular search terms across both periods were “heart arrest” and “heart attack”, with regional variation identified in Osun State, where the term “cardiac arrest” was most frequently searched for in both time periods. Search spikes for the term “cardiac arrest” occurred mainly with celebrity deaths. Awareness and knowledge of cardiopulmonary resuscitation (CPR), cardiac arrest and related terminologies among Nigerian healthcare workers have been noted to be low [27-31] and the levels of awareness and knowledge among non-medical and laypersons in Nigeria remain undocumented. While seeking to determine trends in Google search terms for terms relating to cardiac arrest in the context of a pandemic, spikes and increases in searches for terms including “cardiac arrest” were observed to align with death events which occurred mainly among Nigerian celebrities and politicians, potentially indicating the interest of the Nigerian public for the medical problem behind these events. In particular, the search peak on the 14^th^ of June 2020 corresponded with the death from cardiac arrest of a popular Nigerian celebrity who was a former beauty queen and pastor´s wife, while the search peak on the 21^st^ of July 2020 corresponded with another death from cardiac arrest of a strong political ally and in-law to the sitting Nigerian president.

Deaths from cardiac arrest of two prominent broadcasters, one politician and an ex-Super Eagles player, all from Ogun State, may have contributed to the high searches for the search word “cardiac arrest” in 2020. The increased public interest in certain health conditions has been known to follow celebrity diagnosis [32,33] and celebrity health has been shown to influence public health behavior [34,35]. For instance, Kylie Minogue the Australian-British pop star whose breast cancer illness was publicly discussed by the media and herself, led to the so-called 'Kylie effect' which was characterized by increased breast screening awareness and 40% and 20% increases in mammography and breast screening rates among Australian and British women respectively [36,37]. Actor Charlie Sheen´s 2015 public declaration of his Human Immunodeficiency Virus (HIV) status was associated with 2.75 million internet searches in the United States relating to HIV treatment and prevention [38] while in 2016 American actor, Ben Stiller, twitted about a test that saved his life from prostate cancer, a move that led to the generation of 1.2 million Twitter discussions on cancer [39].

The authors posit that these occasions of the deaths of Nigerian celebrities and politicians present a perfect opportunity, the so-called 'teachable moment', for cardiovascular health promotion. Teachable moments are opportunities created by events or by interactions between individuals to encourage a positive change of habits and positive lifestyle modifications [40,41]. A good case in point is the heart attack suffered by a former President of the United States, Dwight Ike Eisenhower, in 1955 which created a panic among the American populace, especially as CPR was yet to be described in the medical world, heart disease was the leading cause of death at the time, bed rest was the only treatment offered and he was up for re-election later that year [42]. However, the cardiologist engaged to treat Eisenhower was able to allay public fears and anxiety through a series of highly informative press conferences. In a similar context, the Nigerian public can be considered to be most receptive to change when news of Nigerian celebrities and politicians suffering cardiac arrest makes the rounds. Just as celebrity illness announcements made on social media such as Facebook, Twitter and Instagram have been known to increase online searching for health information [39], issues such as prevention and early detection of illness can be facilitated through these media. Another advantage of social media is that one can reach back directly to the audience (e.g. tweet and re-tweet). These celebrity events, the so-called naturally occurring interventions, also present an opportunity to correct a lot of misinformation on health issues which are found on the worldwide web. Thus, government and health authorities can embrace the windows of opportunity presented by these pivotal events to teach the Nigerian public skills on Basic Cardiac Life Support (BCLS) and use of the automated external defibrillator (AED), leading to more favorable outcomes for CA patients as witnessed CA events and prompt bystander CPR have been shown to improve survival rates [43,44].

The public health concern of the respondents in one study conducted in the United States of America has been shown to be far from CA, with topics like cancer, heart disease and diabetes taking first, second and third place respectively in the priority of the respondents [45]. However, when the same respondents were exposed to brief knowledge of CA, interest in CPR and AED was noted to have risen [45]. In October 2018, the International Liaison Committee on Resuscitation (ILCOR) began a global initiative called the World Restart A Heart (WRAH) initiative which aims at increasing public awareness of bystander cardiopulmonary resuscitation (CPR) and improving the rates of bystander CPR and survival from CA globally. This initiative recorded successes in 2019 with the training of over 5.4 million people worldwide in CPR [46]. Nigeria could learn from such initiative by developing local interventions such as the creation of a CPR national awareness day, a day that medical institutions and allied organizations such as the Life Resuscitation Society of Nigeria (LIRESON) and the Nigerian Society of Anaesthetists (NSA) would partner with the Nigerian Orientation Agency (NOA) to roll out public awareness programs to maximize the public health opportunity and ensure that the knowledge of cause of CA and BCLS is widespread in the country. Knowledge of BCLS has the potential to reduce morbidity and mortality following out-of-hospital cardiac arrest (OHCA) [44]. Additionally, CPR-themed Nollywood (Nigerian movie industry) and Kannywood (Hausa language cinema) movies may be impactful in creating awareness for CPR knowledge among laypersons. The influence of broadcast media such as movies and films on public awareness and views of medical issues such as mental health has been known to be significant [47,48].

Individuals who obtain new health information from television talk shows are significantly more health-conscious with more health beliefs and healthy practices than individuals who do not watch such talk shows [49]. Furthermore, BCLS inclusion in Nigerian elementary and secondary school curricula is appropriate and this has been demonstrated successfully in the Flemish Region of Belgium where CPR is embedded within the school curriculum [50]. Mandatory BCLS and first aid classes could be included among the requirements for obtaining a Nigerian driver´s license as practiced in countries like Turkey and Germany [51].

**Limitations**: the increased use of Google Trends™ in medical research highlights the influence of web-based research and big data on the rise in medical infodemiology studies worldwide. Google Trends™ has been shown to be a valid tool for evaluating online search trends of the general public when it concerns health matters [52]. Analyses using Google Trends™ have been applied to a wide variety of areas in medicine including infectious disease transmission, mental health needs assessment [53,54], substance use [55] and non-communicable diseases [33]. While Google Trends™ analysis is thought to be devoid of many of the biases of traditional self-report surveys [53], it has limitations, some of which are reflected in this study. Firstly, the number of searches made was reported by Google Trends™ in relative values and not in absolute values. Furthermore, only members of the public who are English-speaking, those who use Google as their search engine and those with internet access were able to have their data captured, thus excluding people with limited access to or limited skills in the use of the internet or who were non-English speaking. The latter is problematic in Nigeria where more than two-hundred and fifty languages are spoken. In addition, the sample is unknown, precluding the use of data such as sex, age or occupation in the analysis. All these increase the possibility of non-representative sampling bias in the study. Use of specialized medical websites like WebMD® as well as other search engines was not taken into account in this research. Also, it is not possible to tell who is searching and what the intentions of the individual searching might be. However, Google Trends™ has the advantage of being free, easily accessible to the general public, being hosted on the most popular search engine, and being able to report data real-time [56] while capturing public sentiments.

## Conclusion

To the best of our knowledge, this study is the first analysis of information-seeking via the internet for terms relating to cardiac arrest. Findings from this study have provided a window into the mind of the Nigerian public with regards to cardiac arrest and suggest there is an increase in the public web search interest in cardiac arrest during the pandemic compared to the pre-pandemic era although these align with death events of Nigerian celebrities and political figures. Understanding these health information-seeking trends could lead to the development of high-quality and accurate medical content on a website dedicated to the advancement of knowledge of cardiac arrest, CPR and resuscitation created by the Federal Ministry of Health. When hosting such information, content could be optimized to appear in Google searches using search terms highlighted to be popular by this study, for the timely delivery of information to the general public on prevention and management of CA. Finally, celebrity and high-profile deaths generate interest in searches which are potential teachable moments that governments in similar climes could explore educating the general public and sharing information about CA, its causes and possible prevention strategies.

### What is known about this topic

- Awareness and knowledge of cardiac arrest and related terminologies among Nigerian healthcare workers is low and similar awareness and knowledge among the non-medical Nigerian public remains undocumented;
- Internet search queries conducted on Google and reflected by Google Trends™, serve as a proxy for public interest over a period of time;
- With its near real-time performance, Google Trend™ can be used in conjunction with other medical surveillance systems, to improve on the surveillance of medical conditions.

### What this study adds

- Significant increases in searches for “cardiac arrest” and related terms were observed during the COVID-19 pandemic and aligned with death events of Nigerian celebrities and politicians, potentially indicating the interest of the Nigerian public for the medical problem behind these events;
- The occasions of the deaths of the Nigerian celebrities and politicians present a perfect opportunity, the so-called 'teachable moment', for cardiovascular health promotion.

## Annex

**Annex 1**: checklist for documentation of Google trends research, modified from Nuti *et al*. (PDF-84Kb)
